# Multilocus Sequence Typing and Further Genetic Characterization of the Enigmatic Pathogen, *Staphylococcus hominis*

**DOI:** 10.1371/journal.pone.0066496

**Published:** 2013-06-11

**Authors:** Liangfen Zhang, Jonathan C. Thomas, Maria Miragaia, Ons Bouchami, Fernando Chaves, Pedro A. d’Azevedo, David M. Aanensen, Herminia de Lencastre, Barry M. Gray, D. Ashley Robinson

**Affiliations:** 1 Department of Microbiology, University of Mississippi Medical Center, Jackson, Mississippi, United States of America; 2 Laboratory of Molecular Genetics, Instituto de Tecnologia Química e Biológica, Universidade Nova de Lisboa, Oeiras, Portugal; 3 Servicio de Microbiología, Hospital Universitario 12 de Octubre, Madrid, Spain; 4 Universidade Federal de Ciencias de Saude de Porto Alegre, Porto Alegre, Brazil; 5 Department of Infectious Disease Epidemiology, Imperial College London, London, United Kingdom; 6 Laboratory of Microbiology, The Rockefeller University, New York, New York, United States of America; 7 Department of Pediatrics, University of Illinois, Peoria, Illinois, United States of America; University Hospital Münster, Germany

## Abstract

*Staphylococcus hominis* is a commensal resident of human skin and an opportunistic pathogen. The species is subdivided into two subspecies, *S. hominis* subsp. *hominis* and *S. hominis* subsp. *novobiosepticus*, which are difficult to distinguish. To investigate the evolution and epidemiology of *S. hominis*, a total of 108 isolates collected from 10 countries over 40 years were characterized by classical phenotypic methods and genetic methods. One nonsynonymous mutation in *gyrB*, scored with a novel SNP typing assay, had a perfect association with the novobiocin-resistant phenotype. A multilocus sequence typing (MLST) scheme was developed from six housekeeping gene fragments, and revealed relatively high levels of genetic diversity and a significant impact of recombination on *S. hominis* population structure. Among the 40 sequence types (STs) identified by MLST, three STs (ST2, ST16 and ST23) were *S. hominis* subsp. *novobiosepticus*, and they distinguished between isolates from different outbreaks, whereas 37 other STs were *S. hominis* subsp. *hominis*, one of which was widely disseminated (ST1). A modified PCR assay was developed to detect the presence of *ccrAB4* from the SCC*mec* genetic element. *S. hominis* subsp. *novobiosepticus* isolates were oxacillin-resistant and carriers of specific components of SCC*mec* (*mecA* class A, *ccrAB3*, *ccrAB4*, *ccrC*), whereas *S. hominis* subsp. *hominis* included both oxacillin-sensitive and -resistant isolates and a more diverse array of SCC*mec* components. Surprisingly, phylogenetic analyses indicated that *S. hominis* subsp. *novobiosepticus* may be a polyphyletic and, hence, artificial taxon. In summary, these results revealed the genetic diversity of *S. hominis*, the identities of outbreak-causing clones, and the evolutionary relationships between subspecies and clones. The pathogenic lifestyle attributed to *S. hominis* subsp. *novobiosepticus* may have originated on more than one occasion.

## Introduction

Distinguishing commensal from pathogenic coagulase-negative staphylococci (CoNS) is a major challenge for clinical microbiology laboratories. The CoNS are part of the normal bacterial flora of human skin, from which they frequently contaminate clinical specimens, yet they have been increasingly isolated over the past 30 years from human infections associated with indwelling medical devices [1], [2]. *Staphylococcus epidermidis* and *Staphylococcus hominis* are the most common CoNS from the surfaces of the axillae, arms, and legs [3]. *S. hominis* appears to be more successful than other CoNS at colonizing drier regions of the skin such as the volar forearm [4], [5]. Larson et al. [6] reported that *S. hominis* colonization was significantly more frequent on the hands of nurses with damaged skin compared to the hands of nurses with healthy skin. However, *S. hominis* is not limited to colonization of dry environments. Gunn and Colwell [7] reported *S. hominis* to be the most common and phenotypically diverse staphylococcal species isolated from unpolluted marine environments. A link to the marine environment is further supported by the isolation of *S. hominis* from crabs [8] and crabmeat [9].

*S. hominis* can cause nosocomial bloodstream infections [10]–[12] and other opportunistic infections of humans [13], [14]. An increasing proportion of blood culture isolates of *S. hominis* that were atypical or misidentified as *Staphylococcus equorum*, led to the the formal description in 1998 of two *S. hominis* subspecies, including *S. hominis* subsp. *hominis* and *S. hominis* subsp. *novobiosepticus* [15]. Novobiocin resistance and failure to produce acid aerobically from D-trehalose and *N*-acetyl-D-glucosamine are the distinguishing characteristics of *S. hominis* subsp. *novobiosepticus* [15]. Outbreaks and other clusters of bloodstream infections among neonates and adults have been attributed to *S. hominis* subsp. *novobiosepticus* [16]–[19]. However, various difficulties have been encountered in distinguishing between the two subspecies based on standard panels of phenotypes [20], [21], whole-cell protein profiles [22], and sequences from three housekeeping gene fragments [16]. For example, sequence polymorphisms in 16S rRNA and *gyrA* genes were able to distinguish between prototype strains of the two subspecies, but many clinical isolates with *S. hominis* subsp. *hominis* phenotypes were indistinguishable in sequence from isolates with *S. hominis* subsp. *novobiosepticus* phenotypes [16]. Knowledge of the evolutionary relationship of the two subspecies may lead to improved diagnostic tools and an improved understanding of the pathogenic potential of the two subspecies.

Towards this goal, we report the development of a multilocus sequence typing (MLST) scheme based on six housekeeping gene fragments of *S. hominis*. MLST schemes allow bacterial clones to be precisely defined and their relationships to be elucidated at a coarse level [23]. Among staphylococcal species, MLST schemes have been developed for *S. epidermidis*, *Staphylococcus aureus*, *Staphylococcus lugdunensis*, and the *Staphylococcus intermedius* group of species [24]–[27]. However, a recent attempt to develop an MLST scheme for *Staphylococcus haemolyticus*, which may be a close relative of *S. hominis*, concluded that the scheme was of limited use due to the relatively low sequence diversity detected among the isolates [28].

In addition to its occasional pathogenicity, *S. hominis* may be a reservoir of specific components of the methicillin resistance genetic element, SCC*mec*, that may be transferrable to more pathogenic staphylococcal species [29], [30]. Nasal carriage of methicillin-resistant *S. hominis* can be nearly as common as that of methicillin-resistant *S. epidermidis* and *S. haemolyticus* [31]. The relatively high prevalence of methicillin resistance among clinical isolates of *S. hominis*, and especially among *S. hominis* subsp. *novobiosepticus*, has been noted previously [6], [15], [16], [19], [32]. Furthermore, resistance to relatively new antibiotics such as linezolid and quinupristin/dalfopristin has been reported in *S. hominis* [33], [34]. Thus, the ability to genetically characterize and identify relationships between *S. hominis* subspecies and clones will provide a framework for future study of this enigmatic pathogen.

## Materials and Methods

### Bacterial isolates

A total of 108 isolates of *S. hominis* were included in this study. Sixty-eight isolates were from three hospitals in the USA (Illinois, n = 26; Mississippi, n = 1; New York, n = 41), 17 isolates were from one hospital in Spain, including 15 isolates from an outbreak among neonates and two isolates from adults that were not associated with the outbreak [17], five isolates were from a hospital outbreak among adults in Brazil [18], and 16 isolates were selected to be diverse by geography (Argentina, n = 1; Colombia, n = 1; Italy, n = 1; Japan, n = 1; Portugal, n = 2; Mexico, n = 3; Tunisia, n = 7) and by their different *Xho*I pulsed-field gel electrophoresis (PFGE) patterns that were characterized according to Bouchami et al. [30]. In addition, American Type Culture Collection (ATCC) strains ATCC 27844^T^ and ATCC 700236^T^ were included as the *S. hominis* subsp. *hominis* and *S. hominis* subsp. *novobiosepticus* prototype strains, respectively [15]. The isolates were collected from clinical specimens from 1971 to 2011. Ninety-two isolates were from blood cultures, 12 isolates were from other sources such as wound, urine, catheter, and pus specimens, and four isolates were of unknown source. Characteristics of all isolates are listed in Table S1. All isolates were single-colony purified on tryptic soy agar (TSA) plates and stored long-term in tryptic soy broth (TSB) with 15% glycerol at –80°C. Bacterial genomic DNA was extracted with a DNeasy kit (Qiagen, Valencia, USA), according to the manufacturer's instructions.

### Phenotypic characterization

TSA plates with 5% defibrinated sheep blood (Cleveland Scientific, Bath, USA) were used to detect hemolytic activity, and mannitol salt agar (Becton Dickinson, Franklin Lakes, USA) plates were used to detect D-mannitol fermentation, after overnight incubation at 37°C. To detect aerobic acid production from D-trehalose and *N*-acetyl-D-glucosamine, 10 µL loopfuls of bacteria inoculum were streaked 1 cm radially on the surface of purple agar base (Difco, Lawrence, USA) plates that included 1% of either filter-sterilized D-trehalose (Fisher Scientific, Waltham, USA) or *N*-acetyl-D-glucosamine (Alfa Aesar, Ward Hill, USA). These plates were incubated at 35°C and examined at 24 h and 72 h to score acid production in terms of medium-to-strong (+), weak (±), or none (–), as described by Kloos and Schleifer [35]. Briefly, yellow indicator color extends from the culture streak into the surrounding medium within 72 hours for + isolates, yellow indicator color occurs under the culture streak but does not extend into the surrounding medium within 72 h for ± isolates, and very faint to no yellow indicator color occurs under the culture streak within 72 h for - isolates. Each plate was inoculated with up to six testing isolates, plus the positive (ATCC 27844) and negative (ATCC 700236) control strains.

Susceptibilities to novobiocin and oxacillin were determined with disk diffusion tests [36]. Single colonies were used to inoculate 1 mL of TSB, and bacterial cultures were grown to a turbidity comparable to a 0.5 McFarland standard, 100 µl of which was used to inoculate Mueller Hinton agar (Oxoid, Lenexa, USA) plates. The plates were incubated at 35°C for 18 h in the presence of 5 µg novobiocin (Remel, Lenexa, USA) and 1 µg oxacillin (Oxoid) disks. A zone of inhibition <16 mm was defined as novobiocin-resistant as per the manufacturer’s recommendation. A zone of inhibition <18 mm was defined as oxacillin-resistant [36].

### Resistance gene characterization

In staphylococci, resistance to novobiocin can occur by point mutations in *gyrB*, which encodes the target of novobiocin, DNA gyrase B [37]–[39]. Full-length *gyrB* sequences were obtained by primer walking from a diverse set of six novobiocin-sensitive and six novobiocin-resistant isolates. The sequences of both DNA strands of *gyrB* were assembled, edited, and aligned using Lasergene software v7.2.1 (DNAStar, Madison, USA). Nonsynonymous mutations in *gyrB* were compared with the novobiocin susceptibilities to identify candidate resistance mutations. A fragment of *gyrB* containing the only identified candidate resistance mutation was subsequently amplified by PCR in all isolates using the same conditions as used for multilocus sequence typing (described below). These *gyrB* amplicons were subjected to single nucleotide polymorphism (SNP) typing on a Luminex 200 instrument (Millipore, Billerica, USA), according to the protocol outlined in Text S1. PCR primer information is listed in Table S2, and Luminex results for all isolates are listed in Table S3.

Resistance to oxacillin in staphylococci occurs by acquisition of the Staphylococcal Chromosomal Cassette *mec* (SCC*mec*) genetic element [40]. The SCC*mec* element was typed using several PCR assays that score the presence of *mecA* class A, class B, and class C, and *ccrAB1*, *ccrAB2*, *ccrAB3*, and *ccrC* gene complexes [41–43; additional information on the classification of these elements is available at: http://www.sccmec.org]. The presence of the *mecA* gene was scored using a separate PCR assay [43]. Three additional PCR assays, including those of Kondo et al. [43], Oliveira et al. [44], and a modified version of the Oliveira method developed here, were used to score the presence of *ccrAB4*. The modified Oliveira method consisted of decreasing the annealing temperature to 52°C, increasing the number of PCR cycles to 35, and increasing the final elongation time to 10 min. To confirm the specificity of the modified Oliveira method for amplifying *ccrAB4*, amplicons from eight isolates were purified and sequenced on both DNA strands. Alignments of the translated *ccrAB4* sequences along with a variety of reference sequences were made with MUSCLE v3.7 [45] and curated with Gblocks v0.91b [46], using default settings. A maximum likelihood tree was constructed from the curated alignments using PhyML under a WAG model of amino acid substitution [47].

### Multilocus sequence typing (MLST)

A fragment of the *tuf* gene [48] was amplified by PCR and sequenced on both DNA strands for species identification. The *tuf* sequences from all isolates were at least 99% identical to the sequences from one or the other ATCC prototype strains of *S. hominis* used here. The *tuf* gene fragment and six additional housekeeping gene fragments, selected from loci that had been tested for the *S. aureus* and *S. epidermidis* multilocus sequence typing (MLST) schemes [24], [26], were examined for use in a *S. hominis* MLST scheme. These gene fragments were located on separate contigs (ranging in size from ∼52 kb to ∼396 kb) of the unfinished genome sequence of *S. hominis* strain SK119 (GenBank accession number: ACLP00000000.1). The same primers were used for PCR and sequencing on both DNA strands. Thermal cycling conditions for PCR were: 95°C for 2 min; 30 cycles of 95°C for 30 sec, 55°C for 30 sec, and 72°C for 1 min; a final elongation step of 72°C for 2 min. Due to its apparently variable presence among the *S. hominis* isolates, *aroE* was subsequently dropped from the MLST scheme. The following six gene fragments were included in the final MLST scheme: *arcC*, *glpK*, *gtr*, *pta*, *tpiA*, and *tuf*. PCR primer information is listed in Table S2. For each of the six MLST loci, a unique nucleotide sequence defined an allele. Unique allelic profiles, consisting of the allele numbers at each of the six MLST loci, defined sequence types (STs).

### Population genetic and phylogenetic analyses

Nucleotide sequences for each MLST locus were aligned using MUSCLE v3.7 software [45]. The number of polymorphic sites, nucleotide diversity per site (π), and allelic diversity (Hd) were calculated for each locus using DnaSP v5.10 software [49]. ST diversity was measured by Simpson’s index [50], [51]. Multilocus linkage disequilibrium was measured by the standardized index of association (*I*_As_), using LIAN v3.5 software [52].

ClonalFrame v1.2 software [53] was used to estimate population-scaled mutation and recombination parameters. The nucleotide sequence alignments of each MLST locus were input as individual blocks, using the sequences from single representatives of each ST. ClonalFrame was run five times, and each run used a Monte Carlo Markov Chain of 500,000 iterations, discarding the first 250,000 iterations as burn-in and saving every 100th iteration thereafter. The mixing and convergence of the runs were judged to be satisfactory based on inspections with the program's built-in tools. Recombination tract length could not be reliably estimated, so it was fixed to 1000 bp to allow comparisons of other parameter estimates with those of *S. aureus* and *S. epidermidis* [54]. The other parameter estimates represent the averages of the five runs.

To further test the role of recombination in generating allelic variation, the pairwise homoplasy index (PHI) test [55], implemented in SplitsTree v4.0 software [56], was calculated for each locus and for a concatenate of loci. A genetic algorithm for recombination detection (GARD) was used to identify the number and location of recombination breakpoints within each locus [57]. The concatenated nucleotide sequences were also analyzed for reticulate structure by the neighbor-net algorithm [58] implemented in SplitsTree.

### Nucleotide sequences

The *S. hominis* MLST database will be publicly available at shominis.mlst.net. The *ccrAB4* and *gyrB* sequences were deposited in the GenBank database with accession numbers JQ836536-836543 and JQ836544-JQ836555.

## Results and Discussion

### Phenotypic and genetic identification of *S. hominis* subspecies

*S. hominis* subsp. *novobiosepticus* is distinguished from *S. hominis* subsp. *hominis* by novobiocin resistance and failure to produce acid from D-trehalose and *N*-acetyl-D-glucosamine under aerobic conditions [15]. In this study, the disk diffusion tests identified 70 novobiocin-sensitive isolates and 38 novobiocin-resistant isolates. Of the 38 novobiocin-resistant isolates, 37 failed to produce acid aerobically from D-trehalose and *N*-acetyl-D-glucosamine and were classified as *S. hominis* subsp. *novobiosepticus* (Table 1). The remaining 71 isolates were classified as *S. hominis* subsp. *hominis*. As observed previously by Kloos et al. [15], the *S. hominis* subsp. *hominis* isolates were variable in their ability to produce acid aerobically from D-trehalose, *N*-acetyl-D-glucosamine, and D-mannitol (Table 1). As expected for *S. hominis* [15], [59], none of the 108 isolates were hemolytic on blood agar plates.

**Table 1:** Phenotypic and genetic characteristics of *S. hominis* subspecies.

Marker category	Marker subcategory	No. (%) positiveSHN isolates(n = 37)^a^	No. (%) positiveSHH isolates(n = 71)^a^
Subspecies-defining phenotypes	Novobiocin resistance	37 (100)	1 (1)
	D-trehalose	0 (0)	61 (86)
	*N*-acetyl-D-glucosamine	0 (0)	57 (80)
Other phenotypes	D-mannitol	0 (0)	16 (23)
	β-hemolysis	0 (0)	0 (0)
	Oxacillin resistance	37 (100)	46 (65)
Genetic characteristics	*gyrB* allele T431	37 (100)	1 (1)
	*mecA*	37 (100)	46 (65)
	*mecA* class A	37 (100)	42 (59)
	*mecA* class B	0 (0)	4 (6)
	*ccrAB1*	15 (41)	30 (42)
	*ccrAB2*	0 (0)	4 (6)
	*ccrAB3*	22 (59)	1 (1)
	*ccrAB4*	36 (97)	14 (20)
	*ccrC*	29 (78)	21 (30)

a SHN is S. hominis subsp. novobiosepticus, SHH is S. hominis subsp. Hominis.

Examination of full-length *gyrB* sequences from a diverse set of six novobiocin-sensitive and six novobiocin-resistant isolates revealed one SNP, G431T, resulting in a predicted amino acid polymorphism, R144L, that was perfectly associated with the novobiocin susceptibilities (Table 2). This amino acid position may be a hotspot for novobiocin resistance in staphylococci [37]–[39]. A Luminex SNP typing assay was designed for this SNP and was applied to all 108 isolates (Table S3). All 70 novobiocin-sensitive isolates had the G431 allele, and all 38 novobiocin-resistant isolates had the T431 allele. Comparison of the SNP types with the subspecies identification based on the three defining phenotypes, revealed that the SNP typing assay was 100% (37/37) sensitive and 98.6% (70/71) specific for identifying *S. hominis* subsp. *novobiosepticus* (see Table 1). Thus, this SNP typing assay may provide a valid method for subspecies identification after the initial species identification.

**Table 2:** Identification of a candidate novobiocin resistance mutation in *gyrB*.

				Nonsynonymous SNPs and corresponding amino acids in *gyrB* codons^d^								
				137	144	165	220	246	299	336	381	567
Strain	Subspecies^a^	ST^b^	Nov^c^	411	431	493	658	736	896	1006	1141	1700
DAR1263	SHH	1	S	Asp	Arg	Ile	Glu	Ser	Ala	Ile	Ile	Ala
				GAT	CGT	ATA	GAA	TCT	GCA	ATA	ATC	GCT
DAR1286	SHH	10	S									
				...	...	...	...	...	...	...	...	...
DAR1932	SHH	13	S					Pro		Val	Val	
				...	...	...	...	C..	...	G..	G..	...
DAR2030	SHH	1	S									
				...	...	...	...	...	...	...	...	...
DAR3114	SHH	17	S					Pro	Val			Val
				...	...	...	...	C..	.T.	...	...	.T.
DAR3383	SHH	26	S			Val	Lys	Pro		Val		
				...	...	G..	A..	C..	...	G..	...	...
DAR1684	SHN	2	R		Leu							
				...	.T.	...	...	...	...	...	...	...
DAR1919	SHH	8	R	Glu	Leu			Pro				
				..G	.T.	...	...	C..	...	...	...	...
DAR3358	SHN	2	R		Leu							
				...	.T.	...	...	...	...	...	...	...
DAR3374	SHN	16	R		Leu			Pro				
				...	.T.	...	...	C..	...	...	...	...
DAR3377	SHN	2	R		Leu							
				...	.T.	...	...	...	...	...	...	...
DAR3384	SHN	2	R		Leu							
				...	.T.	...	...	...	...	...	...	...

a SHN is S. hominis subsp. novobiosepticus, SHH is S. hominis subsp. Hominis.

b ST is multilocus sequence type.

c Nov is novobiocin susceptibility, S = sensitive, R = resistant.

d In column header, top number is amino acid position, bottom number is nucleotide position; dots indicate identity with the sequence from strain DAR1263.

### Background genetic variation in *S. hominis*

An MLST scheme was developed for *S. hominis* using housekeeping gene fragments from six loci, including *arcC*, *glpK*, *gtr*, *pta*, *tpiA*, and *tuf*. A total of 2,453 bp was sequenced across these six loci for each isolate. One hundred and four SNPs were detected among the isolates, and the number of SNPs per locus ranged from five to 27 (Table 3). Nucleotide diversity, which is the average number of pairwise nucleotide differences per site, ranged from 0.002 to 0.021 for different loci and averaged 0.01 across sequence types (STs) (Table 3). This level of nucleotide diversity is much greater than that recently reported for *S. haemolyticus* (0.00035) [28], and is greater than that recently reported for *S. aureus* and *S. epidermidis* (0.0068 and 0.0064, respectively) [54]. The number of alleles per locus ranged from five to 16, while allelic diversity averaged 0.747 across STs (Table 3). Among all isolates, 40 STs were identified, yielding an ST diversity of 0.887 (0.850, 0.924) by Simpson’s index (95% confidence interval). Among the subset of 16 isolates that were selected to be diverse by geography and their different PFGE patterns (Figure S1), 11 STs were identified, yielding an ST diversity of 0.933 (0.857, 1). These results indicate that *S. hominis* is a genetically diverse species and that this MLST scheme has a relatively high discriminatory power.

**Table 3:** Genetic variation detected by the *S. hominis* MLST scheme.

				All isolates (n = 108)		STs only (n = 40)	
Locus	Sequence length (bp)	No. polymorphicsites (SNPs)	No. alleles	Nucleotide diversity (π)	Allelic diversity (Hd)	Nucleotide diversity (π)	Allelic diversity (Hd)
*arcC*	360	27	16	0.020	0.809	0.021	0.909
*glpK*	435	24	9	0.013	0.729	0.015	0.791
*gtr*	393	20	14	0.006	0.430	0.013	0.792
*pta*	444	13	11	0.004	0.594	0.004	0.731
*tpiA*	450	15	10	0.003	0.565	0.006	0.727
*tuf*	371	5	5	0.003	0.483	0.002	0.533
Average	408.8	17.3	10.8	0.008	0.602	0.010	0.747

### MLST-defined clones of *S. hominis*, including outbreak clones

No STs were shared between the two subspecies. Three STs were identified among the 37 isolates of *S. hominis* subsp. *novobiosepticus*; ST2 (n = 21) was isolated from Argentina, Brazil, Colombia, Spain, and the USA, while ST16 (n = 15) and ST23 (n = 1) were isolated exclusively from Spain. Two sets of *S. hominis* subsp. *novobiosepticus* isolates were from hospital outbreaks. One outbreak was among neonates in Spain [17]; all 15 of these isolates from neonates were ST16, whereas two isolates from adults that were not associated with the outbreak were ST2 and ST23. The isolates from neonates were previously found to be indistinguishable based on PFGE [17]. Another outbreak was among adults in Brazil [18]; four of these isolates were *S. hominis* subsp. *novobiosepticus* ST2, but the fifth isolate was *S. hominis* subsp. *hominis* ST13. The original PFGE data for these Brazilian isolates also showed the fifth isolate to be different from the other four isolates [19]. While the detection of isolate similarities within outbreaks and isolate differences between outbreaks indicates that this MLST scheme may be valid for short-term molecular epidemiological use (i.e. outbreak investigations), some caveats should be noted. First, the outbreaks from Spain and Brazil differed by geography and host age-group. The bacterial clones identified from these two outbreaks may therefore have differed simply because of differences in the geographic or host distributions of these clones. Second, MLST allows for unambiguous, precise identification of clones, but only at a coarse level of detail. In order to distinguish between isolates that have recently diverged from a common ancestor, additional genetic markers may be required. Further studies are needed to determine the extent to which this MLST scheme is appropriate for outbreak investigations.

Thirty-seven STs were identified among the 71 isolates of *S. hominis* subsp. *hominis*; ST1 (n = 26) was the most prevalent ST of this subspecies and was isolated from Mexico, Portugal, Tunisia, and the USA. To our knowledge, S. *hominis* subsp. *hominis* has not been reported to cause outbreaks; however, the relative abundance and wide geographic distribution of ST1 suggests that this subspecies should be monitored more closely. Of note, 81% (30/37) of the *S. hominis* subsp. *hominis* STs were represented by single isolates. Accordingly, ST diversity was significantly higher among *S. hominis* subsp. *hominis*, 0.864 (0.785, 0.943), than among the mostly outbreak-causing *S. hominis* subsp. *novobiosepticus*, 0.527 (0.455, 0.599).

### SCC*mec* variation in *S. hominis*

**Figure 1:**
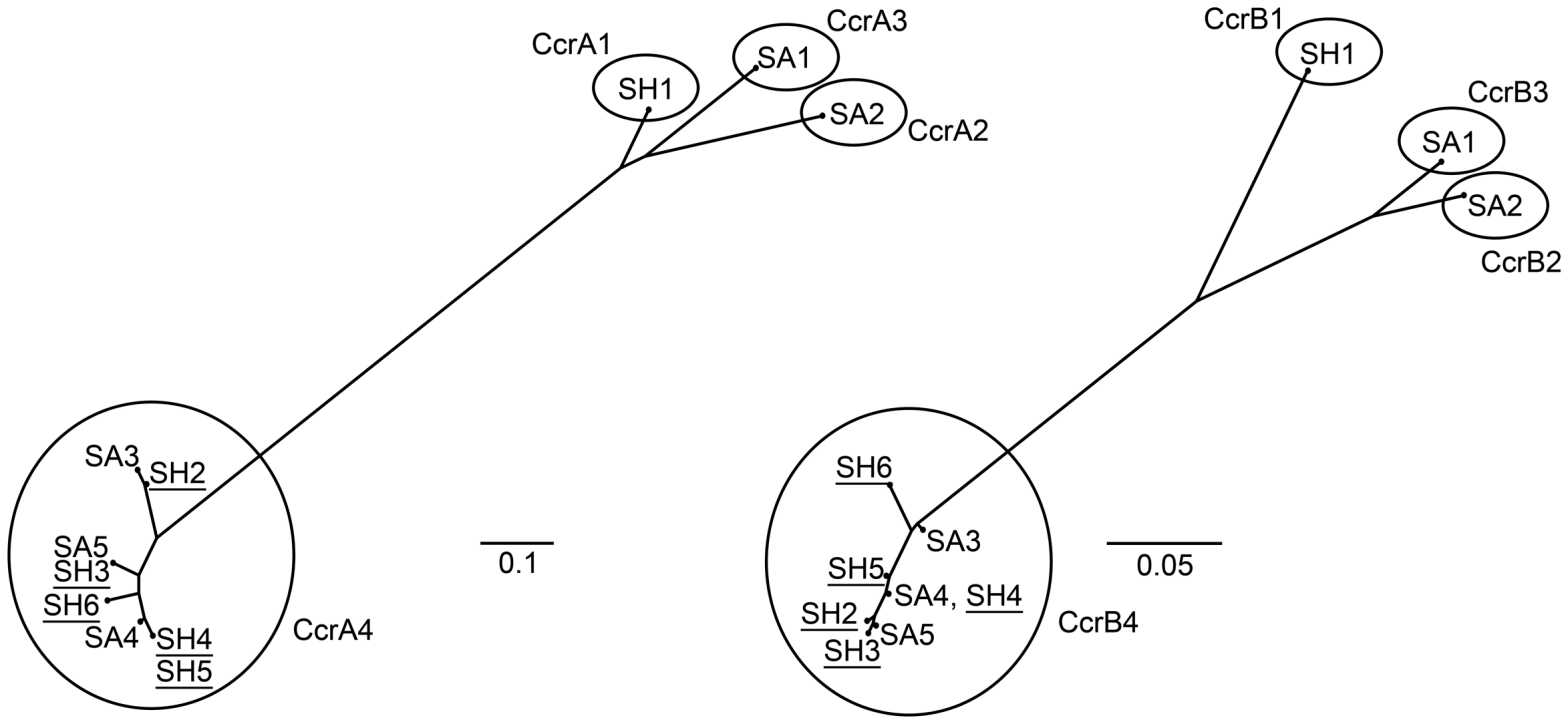
Phylogeny of CcrA4 (panel A) and CcrB4 (panel B) alleles. Translated sequences of *ccrAB* are from the eight *S. hominis* sequences obtained here (SH, underlined), along with various *S. hominis* (SH) and *S. aureus* (SA) reference sequences. Circles outline the major CcrAB alleles. Allelic variant and strain name (and GenBank accession number) are as follows: SH1 = GIFU12263 (AB063171); SH2 = DAR4404 (JQ836542); SH3 = DAR4401 (JQ836541); SH4 = DAR4386 (JQ836536), DAR4388 (JQ836537), DAR4394 (JQ836540), DAR4405 (JQ836543); SH5 = DAR4392 (JQ836539); SH6 = DAR4391 (JQ836538); SA1 = 85-2082 (AB037671); SA2 = N315 (NC_002745); SA3 = HDE288 (AF411935); SA4 = CHE482 (EF126185); SA5 = CHE482 (EF126186).

*S. hominis* has been suggested to be an important source of *mecA* class A, *ccrAB1*, and *ccrAB4*, which are components of some SCC*mec* genetic elements that may be transferrable to *S. aureus* [29], [30]. Two PCR assays for detecting *ccrAB4* have been published [43], [44], but the relative performance of these assays has not been described. We first tested these two PCR assays and a modified version of the Oliveira method, on the 16 isolates that were selected to be diverse by geography and PFGE. The modified Oliveira method yielded an amplicon for 50% (8/16) of these isolates, whereas each of the other two methods yielded an amplicon in single isolates. Phylogenetic analysis of the translated *ccrAB4* sequences from the eight amplicons of the modified Oliveira method revealed these to be genuine *ccrAB4* alleles (Figure 1), which are characteristically divergent from other *ccrAB* alleles [30]. Thus, the modified Oliveira method was determined to be more sensitive than the other two methods and was used to score the presence of *ccrAB4* in the remaining isolates.

All 37 isolates of *S. hominis* subsp. *novobiosepticus* were oxacillin-resistant based on disk diffusion tests and were positive for the *mecA* gene based on PCR (Table 1). SCC*mec* typing of this subspecies revealed three combinations of *ccrAB* alleles with *mecA* class A, including those of SCC*mec* type III (3A, n = 1) and two different nontypeable configurations (3+4A, n = 21; 1+4A, n = 15) (a full listing of SCC*mec* results is in Table S1). *ccrC* was carried by 78% (29/37) of the *S. hominis* subsp. *novobiosepticus* isolates (Table 1).

In contrast, no perfect association between oxacillin susceptibility and carriage of the *mecA* gene was observed for *S. hominis* subsp. *hominis*. Of the 46 isolates that were positive for the *mecA* gene, 45 were oxacillin-resistant and, conversely, of the 25 isolates that were negative for the *mecA* gene, 24 were oxacillin-sensitive; results for these two outlier isolates were confirmed. All isolates that were positive for the *mecA* gene were also typeable for the *mecA* gene complex. Nine isolates had a *ccrAB* complex but no detectable *mecA* gene or gene complex, whereas 11 isolates had no detectable *ccrAB* complex but they had a typeable *mecA* complex. SCC*mec* type I (1B, n = 2), type II (2A, n = 2), type IV (2B, n = 1), type VI (4B, n = 1), and type VIII (4A, n = 4), and four different nontypeable configurations (1A, n = 21; 1+4A, n = 2; 2+4A, n = 1; 3+4A, n = 1) were identified for this subspecies. *ccrC* was carried by 30% (21/71) of the *S. hominis* subsp. *hominis* isolates (Table 1).

Statistical comparisons of the frequency of SCC*mec* components in the two subspecies indicated that *S. hominis* subsp. *novobiosepticus* was a significant source of *mecA* class A, *ccrAB3*, *ccrAB4*, and *ccrC* (Table 1); each *P*<0.0001 in two-tailed Fisher's exact tests. On the other hand, *S. hominis* subsp. *hominis* harbored a more diverse array of SCC*mec* components (Table S1, and described above); Simpson's index of diversity for the different combinations of *mecA*/*ccrAB* complex was 0.527 (0.455, 0.599) for *S. hominis* subsp. *novobiosepticus* and 0.807 (0.727, 0.888) for *S. hominis* subsp. *hominis*, including nontypeable elements but excluding *ccrC* which can be mobilized to locations other than the integration site of SCC*mec* [60]. These subspecies differences in SCC*mec* diversity were somewhat reflected in how strongly STs associated with SCC*mec*. For example, *S. hominis* subsp. *novobiosepticus* ST2 isolates carried the 3A (III) and 3+4A elements and ST16 isolates carried the 1+4A element, whereas the prevalent *S. hominis* subsp. *hominis* ST1 isolates carried five different elements, most of which were nontypeable. The nontypeable SCC*mec* elements identified in this study may represent novel elements, but further work demonstrating that the different components detected by PCR are physically linked is needed to prove this notion. The nontypeable element with *mecA* class A and *ccrAB1* has been reported previously for *S. hominis* [30], [32]; our data show that this element occurs frequently in *S. hominis* subsp. *hominis*, and it occurs together with *ccrAB4* in *S. hominis* subsp. *novobiosepticus*.

### Population genetic signatures of recombination in *S. hominis*

To gain insight into the population structure of *S. hominis*, we first measured multilocus linkage disequilibrium between the MLST loci, using the standardized index of association (*I_AS_*). An *I_AS_* significantly greater than zero indicates a nonrandom association of alleles at different loci. Analysis of all 108 isolates yielded an *I_AS_* of 0.339 (Monte Carlo test with 1000 resamplings, *P*<0.001), and a clone-corrected analysis restricted to single isolates of each of the 40 STs yielded an *I_AS_* of 0.246 (*P*<0.001). Such results are expected for bacterial populations with relatively low recombination rates [61]; however, this index is a crude measure of the impact of recombination on population structure, and linkage disequilibrium can be caused by processes other than rare recombinations [62], [63].

To further examine the role of recombination on *S. hominis* population structure, the MLST nucleotide sequences were analyzed using the Bayesian model implemented by ClonalFrame. As found previously with *S. epidermidis* MLST data, the *S. hominis* MLST data did not allow the recombination tract length parameter to be reliably estimated, so it was fixed to the biologically plausible value of 1000 bp and other parameters were estimated [54]. The per-site mutation rate was based on Watterson's estimator [64], and was the same value as the average nucleotide diversity, 0.01. The per-site recombination rate (95% credibility interval) was estimated to be 0.0016 (0.0009, 0.0025), which does not differ significantly from previous estimates for *S. aureus* and *S. epidermidis* assuming 1000 bp tract lengths (0.0011 and 0.0004, respectively) [54]. However, the estimate of the rate at which nucleotides change by recombinations versus point mutations (95% credibility interval) was 1.73 (1.06, 2.69), which was significantly greater than one and indicative of the greater amount of nucleotide diversity transferred by recombinations in *S. hominis* populations in comparison to those of *S. aureus* and *S. epidermidis* (0.68 and 0.72, respectively) [54]. Consistent with this interpretation, the PHI test detected a signature of recombination (*P*<0.05) within the *glpK* locus. Followup analysis with the GARD algorithm identified a recombination breakpoint (*P*<0.01) at 291 bp into the *glpK* sequence, plus another recombination breakpoint (*P*<0.01) at 177 bp into the *gtr* sequence. Thus, recombination has a significant impact on *S. hominis* population structure.

### Evidence of *S. hominis* subsp. *novobiosepticus* polyphyly

**Figure 2:**
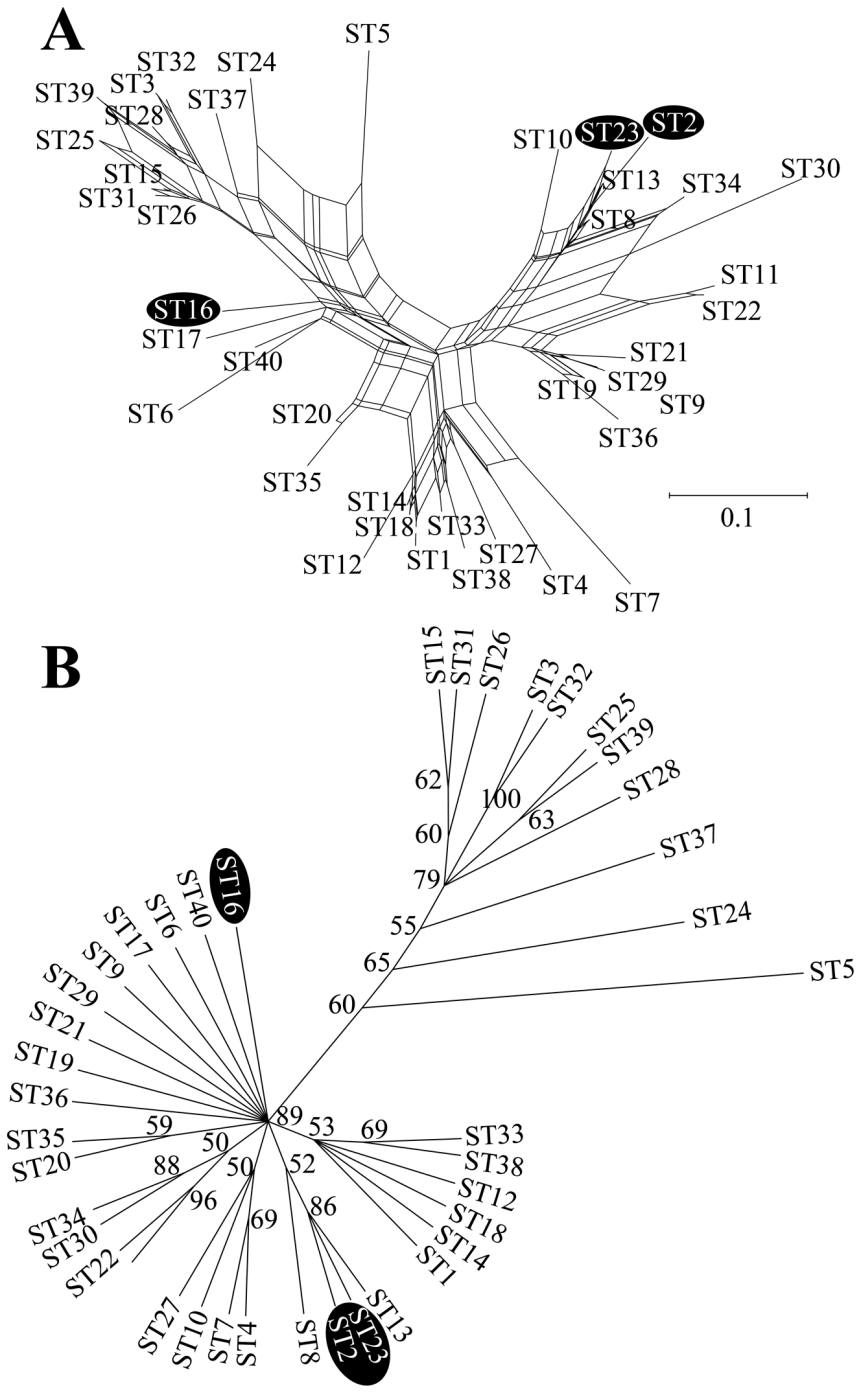
Phylogeny of sequence types (STs) based on neighbor-net (panel A) and ClonalFrame (panel B) algorithms. Numbers at nodes on the ClonalFrame tree are posterior probabilities >50/100. Highlighting indicates the three *S. hominis* subsp. *novobiosepticus* STs.

The three *S. hominis* subsp. *novobiosepticus* STs differed from each other at three to four MLST loci, suggesting that they were not very closely related to each other, whereas the 37 *S. hominis* subsp. *hominis* STs differed from each other at one to six MLST loci. An eBURST analysis of the MLST alleles [65] with default settings showed one major clonal complex of 17 STs, one minor clonal complex of two STs, and 21 divergent STs (Figure S2). A more detailed study of the relationships between STs was made with analyses that accommodate non-treelike patterns, such as those produced by recombination, in the MLST sequences. Analysis with the neighbor-net algorithm revealed a highly reticulate network (Figure 2A). Of note, *S. hominis* subsp. *novobiosepticus* ST16 was separated from ST2 and ST23, suggesting that this subspecies may not be a phylogenetically cohesive group. Also, networks for all of the individual MLST loci except *gtr* separated the *S. hominis* subsp. *novobiosepticus* STs in various combinations (Figure S3). The ClonalFrame tree (Figure 2B) accounts for the influence of recombination on tree branch lengths and topology rather than revealing it in a network, and it provided statistical support for the separation of ST16 from ST2 and ST23.

Interestingly, the single isolate of *S. hominis* subsp. *hominis* ST8, which was the only novobiocin-resistant isolate from this subspecies (Tables 1, 2), clustered with *S. hominis* subsp. *novobiosepticus* ST2 and ST23 (Figure 2A, 2B). In addition, *S. hominis* subsp. *hominis* ST13 was a member of this cluster, and it represented two of the four isolates from this subspecies that failed to produce acid aerobically from D-trehalose and *N*-acetyl-D-glucosamine (the other two isolates were ST4 and ST21; Table S1). As defined by phenotype, ST8 and ST13 were *S. hominis* subsp. *hominis*, but they were more similar to *S. hominis* subsp. *novobiosepticus* by MLST. Thus, this cluster appears to include either evolutionary intermediates or recombinants of the two subspecies.

The phylogenetic separation of the *S. hominis* subsp. *novobiosepticus* STs indicates that this subspecies may be polyphyletic; hence, it may be an artificial taxon whose members have independently gained novobiocin resistance and lost two metabolic traits. Novobiocin resistance may be caused by a single point mutation in *gyrB* (Table 2) [37]–[39], and the two metabolic traits were observed to be variable in this species (Table 1) [15]. These three subspecies-defining phenotypes may be labile from an evolutionary perspective. *S. hominis* subsp. *novobiosepticus* ST16 and ST2 both caused outbreaks, so their obvious pathogenicity may provide a rationale to retain the subspecies designation despite the evidence for their phylogenetic separation. However, if *S. hominis* subsp. *hominis* clones, such as the widespread ST1, were also shown to cause outbreaks, then this rationale for separating the subspecies on the basis of pathogenicity would be less clear.

## Conclusions

*S. hominis* was revealed by MLST to be a genetically diverse species, relative to other staphylococci such as *S. aureus* and *S. epidermidis*, and recombination was shown to have a significant role in generating this diversity. Furthermore, PCR typing of SCC*mec*, using tools adopted from *S. aureus*, yielded a large number of nontypeable elements in *S. hominis*, which is suggestive of novel elements in *S. hominis*. While *mecA* class A, *ccrAB3*, *ccrAB4*, and *ccrC* are significantly more common in *S. hominis* subsp. *novobiosepticus*, a more diverse array of SCC*mec* components are found in *S. hominis* subsp. *hominis*. The two subspecies of *S. hominis* are distinguished by three phenotypes, but phylogenetic analyses indicate that *S. hominis* subsp. *novobiosepticus* STs do not form a single, well-supported cluster to the exclusion of all other STs; that is, the subspecies may be a polyphyletic, artificial taxon. Future genomic investigations of the *S. hominis* clones identified here should provide more insight into the nature of the subspecies and the role of recombination in generating the apparent polyphyly.

## Supporting Information

Figure S1 **Pulsed-field gel electrophoresis (PFGE) patterns and sequence types (STs) for 16 isolates selected to be diverse by geography and PFGE patterns.**
(PPT)

Figure S2 **Relationships between sequence types (STs) as inferred from the eBURST algorithm with default parameters.** Each dot represents a different ST. Lines indicate that STs differ at one of the six loci used for multilocus sequence typing.
(TIF)

Figure S3 **Neighbor-net networks for each of the six loci used for multilocus sequence typing.**
(TIF)

Table S1 **Characteristics of study isolates.**
(XLS)

Table S2 **Additional primer sets.**
(DOC)

Table S3 **Results for Luminex SNP typing of** ***gyrB*** **.**
(XLS)

Text S1 **Supplemental methods.**
(DOC)
