# Supplementary material for: Multilocus Sequence Typing and Further Genetic Characterization of the Enigmatic Pathogen, Staphylococcus hominis
Source: PLoS One. 2013 Jun 11;8(6):e66496. doi: 10.1371/journal.pone.0066496 (PMC3679023; doi:10.1371/journal.pone.0066496)
Supplement: Figure S1 — Pulsed-field gel electrophoresis (PFGE) patterns and sequence types (STs) for 16 isolates selected to be diverse by geography and PFGE patterns. (PPT) [file pone.0066496.s001.ppt]

## Slide 1
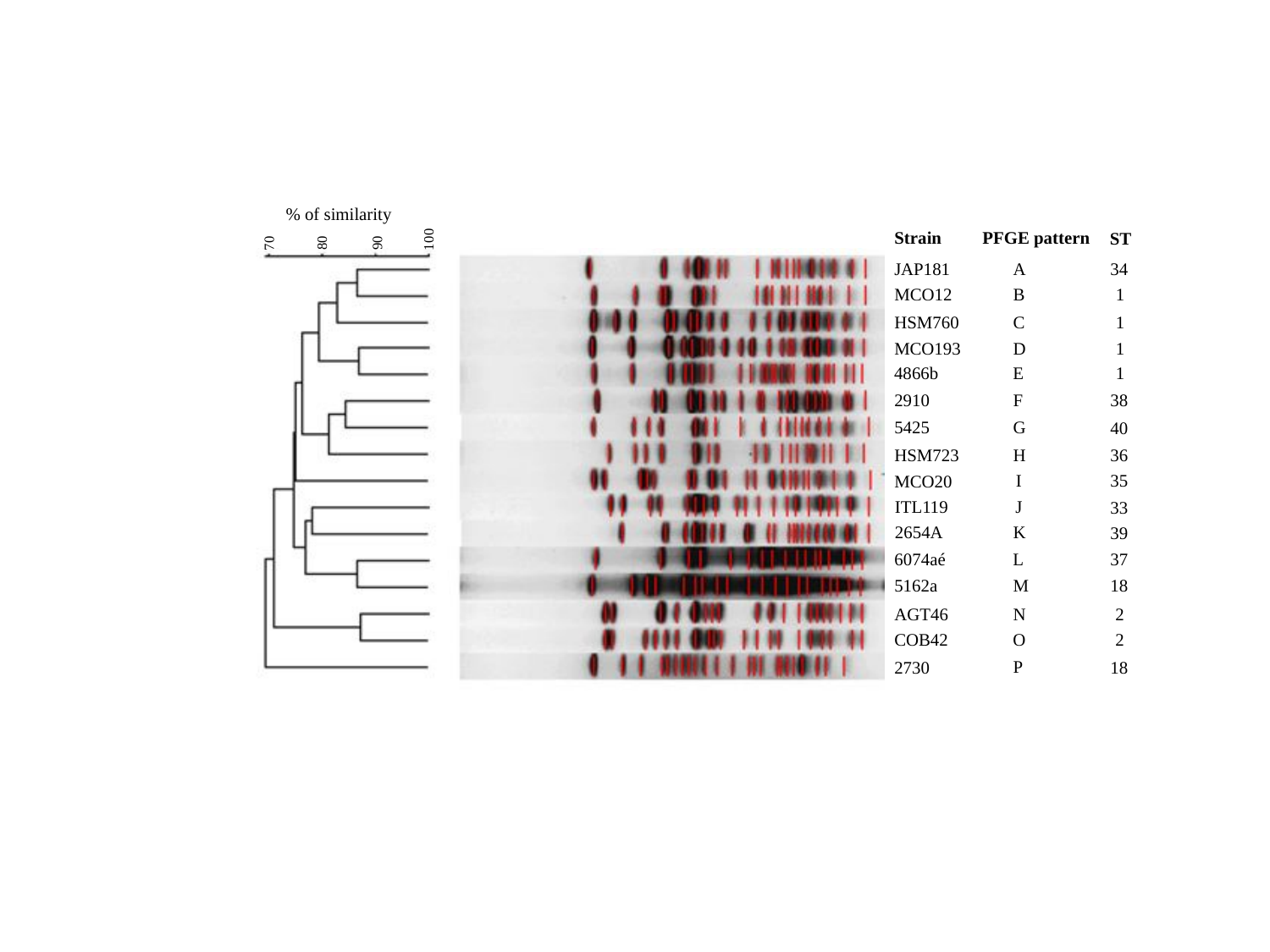

% of similarity
80
90
70
100
Strain
PFGE pattern
ST
A
JAP181
34
MCO12
B
1
HSM760
C
1
MCO193
D
1
4866b
E
1
2910
F
38
5425
G
40
HSM723
H
36
I
35
MCO20
ITL119
J
33
2654A
K
39
L
37
6074aé
M
5162a
18
AGT46
N
2
O
COB42
2
P
2730
18
