# Supplementary figures and images for: Multilocus Sequence Typing and Further Genetic Characterization of the Enigmatic Pathogen, Staphylococcus hominis
Source: PLoS One. 2013 Jun 11;8(6):e66496. doi: 10.1371/journal.pone.0066496 (PMC3679023; doi:10.1371/journal.pone.0066496)

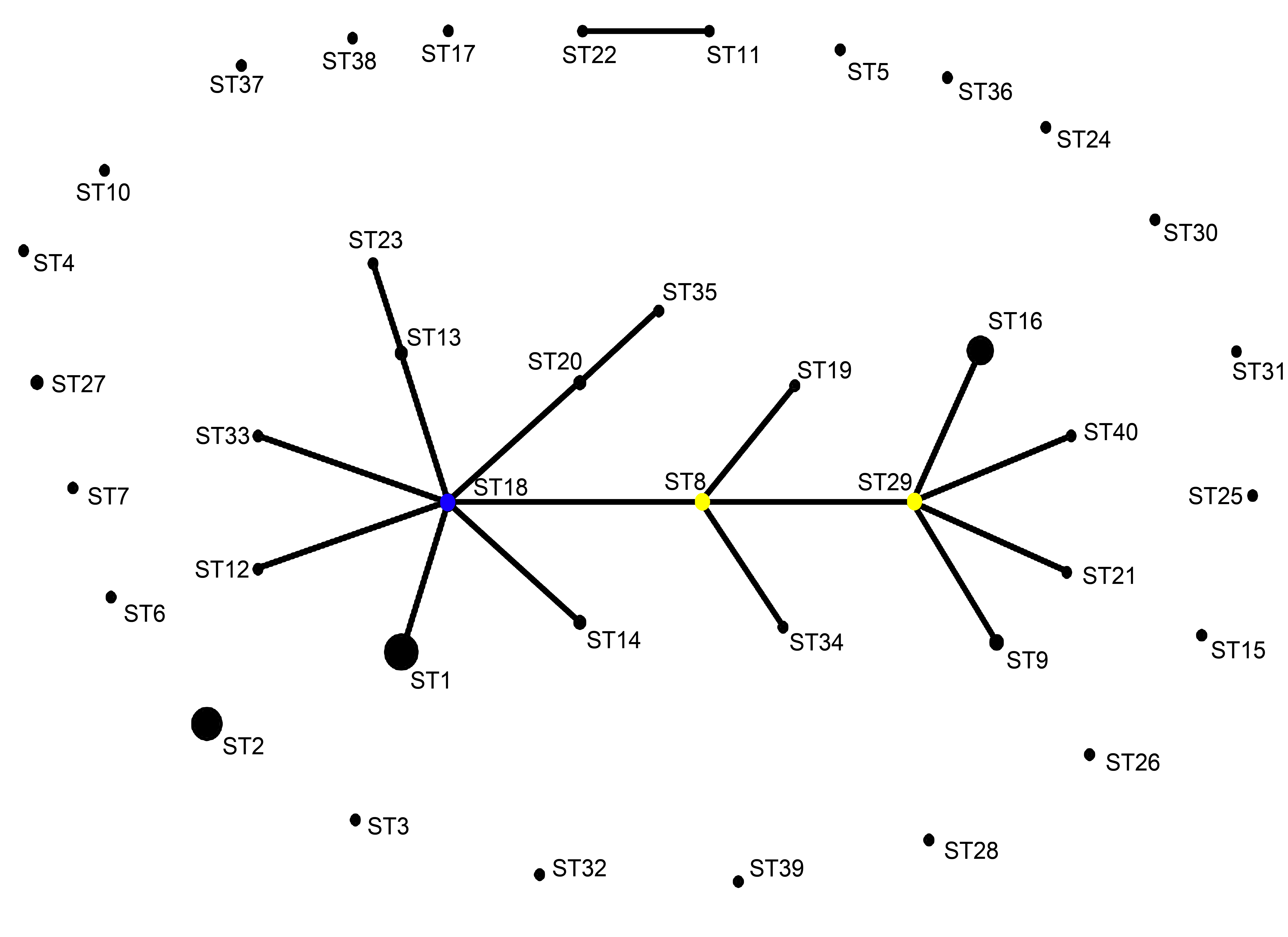

Supplement: Figure S2 — Relationships between sequence types (STs) as inferred from the eBURST algorithm with default parameters. Each dot represents a different ST. Lines indicate that STs differ at one of the six loci used for multilocus sequence typing. (TIF) [file pone.0066496.s002.tif]

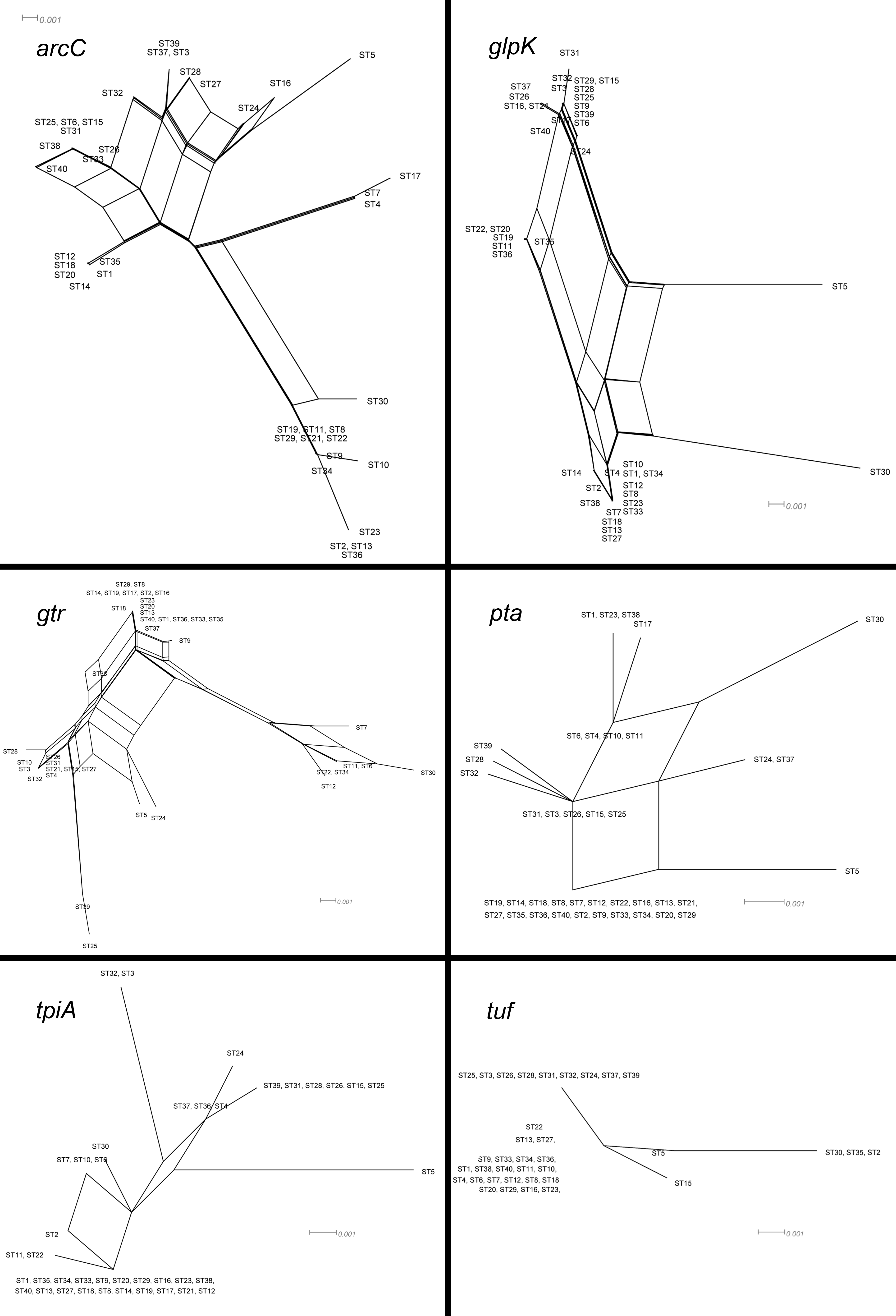

Supplement: Figure S3 — Neighbor-net networks for each of the six loci used for multilocus sequence typing. (TIF) [file pone.0066496.s003.tif]
