# Supplementary material for: Multilocus Sequence Typing and Further Genetic Characterization of the Enigmatic Pathogen, Staphylococcus hominis
Source: PLoS One. 2013 Jun 11;8(6):e66496. doi: 10.1371/journal.pone.0066496 (PMC3679023; doi:10.1371/journal.pone.0066496)
Supplement: Table S2 — Additional primer sets. (DOC) [file pone.0066496.s005.doc]

| **Primer**  **name** | **Forward (5’-3’)** | **Reverse (5’-3’)** | **Use** | **Amplicon Length (bp)** |
| --- | --- | --- | --- | --- |
| SHarcC | TCGCGTTGTGCCCTCTCC | ATTTTACCTTCTAGCGCATCATTT | MLST | 441 |
| SHglpK | GGCAATCTCGTCAAACACAACAT | CCATACGTATTTTTCACATCACCA | MLST | 490 |
| SHgtr | GTTGTCACATTAATTGGTCGTTCC | ATTTACAAGTTCAGGGTCAAGTGC | MLST | 438 |
| SHpta | GTCCGTCCTGCCTTACAAA | CAATCGCTTCAAATCCACCTA | MLST | 484 |
| SHtpiA | TGGTGCATATACAGGAGAAACTT | TGATGCGCCACCAACTAA | MLST | 499 |
| tuf | GCCAGTTGAGGACGTATTCT | CCATTTCAGTACCTTCTGGTAA | MLST and species identification | 412 |
| SHgyrB1 | CCGATTTTATTATTAGATGATGTC | CTCTAATACGTTTTTGAAGTGTTT | *gyrB* sequencing and SNP typing | 793 |
| SHgyrB2 | TGGTGTAGGTTCATCTGTAGTAAA | GATTCTCATATAAAAAGCGTTCAA | *gyrB* sequencing | 755 |
| SHgyrB3 | GTGAAGGTTTAACAGCAGTCGTCT | TTTTGGAGTAGGATTTAGTTCAGC | *gyrB* sequencing | 743 |
| SHgyrB4 | TGTATACATCGCTCAACCACCAT | TCAACAAGAGGATAACGATAGC | *gyrB* sequencing | 686 |

*tuf* primers are from Heikens et al. (2005)
