# Supplementary material for: Multilocus Sequence Typing and Further Genetic Characterization of the Enigmatic Pathogen, Staphylococcus hominis
Source: PLoS One. 2013 Jun 11;8(6):e66496. doi: 10.1371/journal.pone.0066496 (PMC3679023; doi:10.1371/journal.pone.0066496)
Supplement: Text S1 — Supplemental methods. (DOC) [file pone.0066496.s007.doc]

**Protocol for Luminex SNP typing of *gyrB***

To remove excess PCR primers and dNTPs, 10 µl of PCR products were treated with 1 µl of an enzyme mixture containing 5 U of exonuclease I (Exo) and 0.5 U of shrimp alkaline phosphatase (SAP) (Fisher Scientific) at 37°C for 30 min, followed by 20 min at 80°C to inactivate the enzymes. The allele-specific primer extension (ASPE) primers, MTAG056gyrB419W, 5’-CTT AAA CTC TAC TTA CTT CTA ATT TGA TGA TAA ATT GTT TCA TTA C and MTAG043gyrB419M, 5’-AAC TTT CTC TCT CTA TTC TTA TTT TGA TGA TAA ATT GTT TCA TTA A, were designed manually to target the wild-type (novobiocin-sensitive) and mutant (novobiocin-resistant) alleles by including allele-specific nucleotides at the 3’ end of each primer. At the 5’ end, reverse complements of the anti-TAG sequences (24-mer) of MagPlex®-TAG^TM^ microspheres (Luminex) MTAG-A056 and MTAG-A043 were included. Each 20 µl ASPE mixture reaction contained 5 µl Exo-SAP treated PCR product, 25 nM of each ASPE primer, 0.75 U of *Tsp* DNA polymerase (Invitrogen, Carlsbad, USA), 1x buffer (supplied with *Tsp* polymerase), 5 µM dATP/dGTP/dTTP and biotin-dCTP (Invitrogen) and 1.25 µM MgCl_2_. Thermal cycling was done on a MyCycler (Bio-Rad, Hercules, USA) at 95°C for 5 min, followed by 30 cycles of 94°C for 30s, 55°C for 30 s and 72°C for 1 min, and a final extention at 72°C for 3 min.

A microsphere mixture containing 100 microspheres/µl for each of MTAG-A056 and MTAG-A043 was prepared in 2x Tm hybridization buffer (0.4 M NaCl/0.2 M Tris/0.16% Triton X-100, pH 8.0). 5 µl of microsphere mixture was added to a final 50 µl hybridization reaction consisted of 10 µl of ASPE product, 20 µl of 2x Tm hybridization buffer and 15 µl of dH_2_O. A microsphere control contained 5 µl of microsphere mixture, 20 µl of 2x Tm hybridization buffer and 25 µl of dH_2_O, while a blank control contained 20 µl of 2x Tm hybridization buffer and 30 µl of dH_2_O. Hybridization was done on a MyCycler (Bio-Rad) at 95°C for 90 s, followed by 37°C for 30 min. The hybridized microspheres were subsequently pelleted by centrifugation, washed twice with 1x Tm hybridization buffer, finally resuspended in 75 µl 1x Tm hybridization buffer containing 2 ng/ml streptavidin-R-phycoerythrin (Invitrogen) for each well then incubated at 37°C for 15 min. Fifty µl of the reaction was loaded into a 96 well V-bottom PCR plate and run through a Luminex 200 instrument with xPONENT v3.1 software for SNP calling. The xPONENT software calculates the net median fluoresence intensity (MFI) of both alleles by subtracting the MFIs of microsphere controls and blank controls, and it calculates the proportions of both alleles (MFI_called allele_/MFI_wild-type allele_+MFI_mutant allele_). Based on the MFIs and allelic proportions of the 12 isolates with full-length *gyrB* sequences, described in the text, a SNP was called when the net MFI was >300 and the allelic proportion was >0.85 (data presented in Table S3).
